# Hormonal Regulation of Sertoli and Leydig Cell States in Non-Obstructive Azoospermia: A Critical Narrative Review

**DOI:** 10.3390/cimb48100970

**Published:** 2026-09-22

**Authors:** Aris Kaltsas, Eleftheria Markou, Maria-Anna Kyrgiafini, Zissis Mamuris, Michael Chrisofos

**Affiliations:** 1 Third Department of Urology, Attikon University Hospital, School of Medicine, National and Kapodistrian University of Athens, 12462 Athens, Greece; mchrysof@med.uoa.gr; 2 Department of Microbiology, University Hospital of Ioannina, 45500 Ioannina, Greece; eleftheria.markou@uoi.gr; 3 Laboratory of Genetics, Comparative and Evolutionary Biology, Department of Biochemistry & Biotechnology, University of Thessaly, 41500 Larissa, Greece; mkyrgiafini@uth.gr (M.-A.K.); zmamur@bio.uth.gr (Z.M.)

**Keywords:** non-obstructive azoospermia, Sertoli cells, Leydig cells, testicular somatic niche, somatic-cell maturation, androgen receptor, gonadotropins, single-cell RNA sequencing

## Abstract

Non-obstructive azoospermia (NOA) includes diverse disorders whose somatic-cell abnormalities depend on developmental history, endocrine exposure, genotype, and tissue injury. This critical narrative review examines hormonal regulation of Sertoli and Leydig cell states and the interpretation of maturity-associated findings in human NOA. PubMed searching and reference tracing informed a purposive synthesis of developmental, histological, molecular, and interventional evidence. Human studies describe heterogeneous immature-like programs, spatially restricted mature features, and injury-associated dysfunction. Fetal Klinefelter findings indicate early somatic abnormalities, whereas adult tissue studies reveal local differences in chromosome constitution and germ-cell support. These observations address distinct biological questions and should be interpreted within their original designs. The proposed framework records maturity-associated evidence, injury, and chronology separately within each somatic compartment. Convergence across molecular and non-transcriptomic domains can strengthen maturity characterization, but the suggested reporting criterion remains unvalidated. Serial-biopsy evidence in severe congenital hypogonadotropic hypogonadism supports hormone-dependent forward Sertoli maturation. Its applicability to primary testicular NOA remains uncertain. Cross-sectional and ex vivo findings identify candidate mechanisms but do not establish within-person reversal or validated sperm-retrieval prediction.

## 1. Introduction

Non-obstructive azoospermia (NOA) is a clinical endpoint with genetic, developmental, endocrine, and acquired causes [1,2]. The World Health Organization (WHO) laboratory manual defines azoospermia by the absence of spermatozoa, including examination of sediment from a centrifuged ejaculate [3]. This laboratory finding does not distinguish obstructive from non-obstructive disease. Confirmation and etiological classification require repeat semen assessment and an andrological evaluation [4]. Large sequencing cohorts have identified monogenic subforms within apparently idiopathic spermatogenic failure [5].

Histological patterns include Sertoli-cell-only (SCO), germ-cell maturation arrest, hypospermatogenesis, and tubular sclerosis. These patterns may coexist or differ between sampling sites [6]. Histology records germ-cell progression and tissue architecture, but the sampled endpoint does not reconstruct the sequence that produced it. This distinction defines the inference boundary of a cross-sectional testicular biopsy (Figure 1).

Serum hormones, testicular volume, and histology inform diagnosis and counseling, but incompletely predict surgical sperm retrieval [7,8]. They also leave a biological question unresolved: did the somatic compartment fail to mature, change after injury, or retain mature features while losing function?

Previous reviews have summarized somatic-cell states and discussed blocked Sertoli maturation in NOA [9]. This review examines how hormonal history shapes those states and how molecular, structural, and functional observations support different interpretations. Its scope includes Sertoli and Leydig biology, their local interactions, and the distinction between maturity-associated phenotype, tissue injury, and chronology. Developmental endocrine disorders and acquired hormonal suppression provide mechanistic comparators; clinical hormonal pretreatment before sperm retrieval is outside the main scope.

## 2. Review Approach and Terminology

### 2.1. Literature Sources and Scope

A critical narrative approach was used because the question spans developmental biology, disease mechanisms, and the interpretation of heterogeneous experimental designs. PubMed provided the database search, covering inception through 15 August 2026. The recorded query combined Medical Subject Headings and free-text terms for NOA, somatic-cell development, endocrine regulation, cellular plasticity, and single-cell or spatial methods. The search log records 644 results from this single combined query, rather than a sum of separate keyword searches. Developmental, comparator, animal, and ex vivo evidence was also identified through reference lists of retrieved articles and relevant reviews. PubMed was the sole database used, and its NOA-centered query did not comprehensively retrieve these broader topics. The executable query, limits, and selection rationale are retained in the Supplementary Table S1 Search Strategy. Selection prioritized relevance to hormonal regulation and the interpretation of somatic-cell states. Human evidence was preferred; animal and ex vivo studies supplied mechanistic context. Studies in Supplementary Tables S2 and S3 were selected to illustrate different etiologies and designs, without exhaustive eligibility accounting. No systematic screening flow or quantitative risk-of-bias score was produced. The Scale for the Assessment of Narrative Review Articles (SANRA) criteria informed the narrative review, including transparent description of the search and evidence [10].

### 2.2. Clinical and Biological Terminology

NOA denotes a clinical condition; SCO and maturation arrest describe tissue patterns [11,12]. Germ-cell maturation arrest does not by itself indicate somatic-cell immaturity. Table 1 consolidates the biological terms and the proposed evidence descriptors used in this review. Molecular resemblance to an earlier developmental state is distinguished from a broader maturity characterization and from claims about persistence or acquisition.

The distinction between developmental immaturity and later change predates current single-cell methods. Steger et al. described contrasting marker patterns with and without retained prepubertal-type cords, interpreted as maturation block or reversion of differentiated features [13]. Those interpretations remain relevant, while the chronology of a human cellular state requires evidence beyond a cross-sectional pattern. Primary and secondary SCO similarly express proposed developmental histories. In a series of 41 men, their histological distinction had limited value for predicting sperm detection [14].

### 2.3. Interpretation of Evidence

Each study was considered in relation to its original question and its contribution to this review. Interpretation considered temporal design, support for causation, independent donor replication, validation across methods, functional readouts, and applicability to primary testicular NOA. These features were not combined into a score. Developmental endocrine disorders, acquired suppression, aging, and experimental systems were distinguished from direct primary-NOA evidence. Condition-level findings and selected study examples are tabulated where they are discussed. The fuller extraction is provided in Supplementary Table S2.

The proposed reporting criterion was developed during this synthesis and has not been validated as a diagnostic or study-quality instrument. It describes additional evidence that could strengthen a maturity interpretation, rather than judging whether a study fulfilled its original purpose. Unmeasured features were recorded as not assessed. Evidence extraction was performed by A.K. All 15 study-by-compartment entries were then verified against the primary sources by a second author (E.M.), who recorded the reported evidence, its location, the supported inference, and the available temporal information. Three source descriptions were corrected as a result. This verification checked the recorded evidence rather than repeating the classification without sight of it, so no inter-rater agreement statistic is reported. Selection and interpretation therefore remain subject to author judgment.

The figures were prepared with BioRender AI from author-written specifications and reviewed for scientific content.

## 3. Normal Development of Sertoli–Leydig Cell States

Sertoli and Leydig states develop through fetal specification, minipuberty, childhood remodeling, and asynchronous pubertal maturation. Each window combines hormonal exposure with changing receptor competence and tissue architecture. Adult phenotypes therefore reflect the cumulative effects of these processes and germ-cell–somatic interactions, rather than current gonadotropin or testosterone concentrations alone (Figure 2).

### 3.1. Fetal Specification and Testicular Niche Formation

At approximately 6 postconceptional weeks (PCWs), sex-determining region Y (*SRY*) expression initiates testicular development, and SOX9-positive supporting cells acquire a fetal Sertoli state (Figure 2A). These cells organize primordial germ cells into cords and secrete anti-Müllerian hormone (AMH) and paracrine signals, including desert hedgehog (DHH). A single-cell multi-omic and spatial atlas examined 22 human male gonads at 6–21 PCW. It placed early supporting states between GATA-binding protein 4 (GATA4)-positive coelomic epithelium and Sertoli states, with Leydig, vascular, immune, and interstitial populations around the cords [15]. The cords separate the germ-cell-supporting compartment from the androgen-producing interstitium while maintaining reciprocal signaling between them.

A complementary atlas of approximately 32,500 embryonic, fetal, and infant testicular cells identified divergent Sertoli and interstitial/Leydig programs between weeks 7 and 8. Both emerged computationally from a heterogeneous somatic progenitor-like population present at weeks 6–7. Sertoli cells expressed structural and tube-forming programs; Leydig cells acquired steroidogenic genes, with interstitial CYP17A1 protein detected from week 7. Germ cells began acquiring fetal spermatogonial features around weeks 14–16, after somatic niche specification. Tissue localization supports these developmental associations, while pseudotime proposes their order [16]. These complementary atlases resolve coordinated cell-state diversification while leaving the proposed lineage relationships open to experimental testing.

Fetal Leydig cells produce androgens for masculinization and insulin-like factor 3 (INSL3), which is detectable soon after testis determination and supports the first phase of testicular descent. Early human steroidogenesis depends mainly on placental human chorionic gonadotropin (hCG), followed by fetal luteinizing hormone (LH). INSL3 reflects differentiated Leydig function more constitutively than acutely regulated testosterone synthesis [17,18]. Fetal Sertoli cells express SOX9, AMH, KRT8/18, and the follicle-stimulating hormone (FSH) receptor (FSHR). Their androgen-receptor (AR) expression is minimal or absent, and the cords lack an adult blood–testis barrier.

### 3.2. Minipuberty and Childhood

Withdrawal of placental steroids permits transient hypothalamic–pituitary–gonadal activation after birth (Figure 2B). During minipuberty, within the first 3–6 months, LH-driven testosterone and FSH-associated inhibin B rise alongside age-dependent changes in proliferation and apoptosis in germ, Sertoli, and interstitial cells. Leydig activity also expands during this transient endocrine stimulation. In 44 prepubertal testes, newborn growth was associated more strongly with reduced apoptosis than increased proliferation, consistent with remodeling before the postnatal testosterone peak [19]. Inhibin B remains detectable throughout childhood despite low circulating testosterone, indicating continued seminiferous activity [20].

Minipuberty does not reproduce puberty. Fetal and neonatal Sertoli cells express *FSHR* but little or no AR, allowing testosterone to coexist with high AMH. Human tissue, androgen-insensitivity phenotypes, and an AR-expressing Sertoli-cell model link AR competence to testosterone-mediated *AMH* repression [21]. FSH can therefore support Sertoli expansion and inhibin production before adult androgen responsiveness develops. Meiosis additionally depends on germ-cell competence and tissue organization, so early androgen production alone cannot initiate the pubertal program.

Childhood changes extend beyond the minipubertal hormone surge (Figure 2C). In 48 human testes spanning 7 PCW to 13.5 years, Sertoli proliferation peaked at 2–2.5 years and declined through early childhood and became minimal from approximately 6 years. AMH decreased and AR increased between 4 and 10 years [22]. Small lumina appeared in some specimens near 6.5 years [22]. By contrast, a four-donor atlas found no clear lumen at age 7 and progressive tubulation from age 11 [23]. Sparse sampling and differing morphological criteria may contribute to this variation in the observed timing of lumen formation.

### 3.3. Pubertal Sertoli Maturation

Pubertal reactivation of pulsatile gonadotropin-releasing hormone (GnRH) increases LH and FSH (Figure 2D). Leydig steroidogenesis raises intratesticular testosterone, while FSH supports Sertoli metabolism and germ-cell-supporting capacity. Acquisition of functional Sertoli AR permits androgen-dependent *AMH* repression, cell-cycle withdrawal, basal nuclear positioning, polarization, and junctional remodeling. Completion of blood–testis barrier organization, germ-cell progression, and spermiation also require competent germ cells and coordinated paracrine, peritubular, and architectural inputs [24,25].

An age-stratified study of 84 control specimens linked Sertoli AR acquisition, AMH loss, and connexin 43 expression to successive developmental stages [26]. Sustained AR staining appeared between 4.6 and 10.8 years, following rare weak minipubertal staining. AMH was no longer detected between 9.2 and 10.2 years; connexin 43 and spermatogenesis appeared in older specimens, between 10.8 and 13.8 years. Four boys with complete androgen insensitivity retained high AMH without connexin 43 or spermatogenesis. Conversely, four childhood Leydig-cell tumors were associated with locally advanced Sertoli maturation and spermatogenesis [26]. These comparisons connect androgen competence and local exposure with tissue organization. The age ranges describe cross-sectional specimens rather than within-individual transitions.

The four-donor pubertal atlas profiled approximately 10,000 cells from boys aged 7, 11, 13, and 14 years. Two prepubertal Sertoli transcriptional states computationally converged toward a mature program, with increasing AR targets, morphogenesis, extracellular matrix (ECM), and innate-immune genes and declining *AMH*. Immunostaining supported AR gain, AMH loss, and tubular reorganization [23]. Adult single-cell atlases provide complementary molecular references [27,28]. Developmental ECM and immune programs require contextual interpretation because disease-associated remodeling can involve the same pathways.

### 3.4. Leydig Differentiation and Sertoli–Leydig Coupling

Leydig steroidogenic activity declines after minipuberty, but the relationship between human fetal, transitional, and adult populations remains unresolved. In 33 fetal/prepubertal and 58 adult pathological specimens, DLK1 predominated in fetal and spindle-shaped progenitor-like Leydig cells; INSL3 marked most differentiated adult Leydig cells. CYP11A1 appeared across more stages, limiting its discriminatory value [29]. The adult specimens were pathological, with no normal adult control group; the DLK1–INSL3 contrast therefore describes phenotypes within that sampling context. The pubertal atlas inferred separation of a shared prepubertal Leydig/myoid program into steroidogenic Leydig and contractile peritubular states [23]. Mature Leydig phenotypes combine STAR, CYP17A1, HSD17B3, and INSL3 expression with hormone-responsive steroidogenesis; peritubular cells form the contractile, ECM-rich tubular wall (Figure 2E). Fetal and adult populations share several of these steroidogenic markers, so marker positivity alone does not establish an adult stage [29].

Endocrine trajectories also show asynchronous development. In a five-year longitudinal pilot study of ten healthy boys, LH rose before INSL3 in six and at the same examination in four. LH, testosterone, and INSL3 did not increase in a fixed order [30]. INSL3 thus complements testosterone when evaluating Leydig differentiation, while Sertoli AR competence determines the response to the available androgen. Signals from Sertoli, germ, peritubular, and immune cells also contribute to Leydig and tubular organization. Development thus coordinates endocrine activity with cellular responsiveness and assembly of the seminiferous niche.

## 4. Hormonal and Paracrine Regulation of Somatic-Cell States

FSH-dependent Sertoli expansion, LH/hCG-dependent steroidogenesis, and Sertoli AR competence connect hormonal exposure to developmental responses (Figure 3). Paracrine signals modify these responses through intracellular growth pathways and communication between neighboring cells. Human developmental and monogenic evidence defines several components; animal perturbations establish mechanisms that remain less directly tested in human NOA. A pathway required for normal maturation may be causal, compensatory, or associated with tissue damage in primary NOA. Its developmental role alone cannot determine which interpretation applies.

### 4.1. FSH–FSHR and Sertoli Expansion

FSH expands the proliferating Sertoli population and increases eventual germ-cell-supporting capacity. It also promotes AMH and inhibin B secretion, metabolism, and survival. In eight untreated adult men with congenital hypogonadotropic hypogonadism (CHH), one month of recombinant FSH (rFSH) increased AMH and inhibin B without changing LH or testosterone. Seven subsequently received rFSH plus hCG; rising testosterone accompanied AMH suppression despite continued FSH stimulation [31]. This intervention demonstrates sequential biochemical responses to FSH and added LH-like activity. Structural terminal maturation was not assessed, making this a demonstration of hormonal responsiveness within an initially immature compartment.

FSHβ-null male mice remained fertile despite smaller testes and reduced sperm output, separating quantitative spermatogenic capacity from qualitative completion in this model [32]. Combined loss of *FSHR* and Sertoli AR reduced Sertoli number by approximately half and left only a small fraction of control germ-cell numbers. The combined defect caused more severe early meiotic failure than either isolated defect [33].

Thyroid hormone constrains the expansion window in rodents. Sertoli-specific expression of dominant-negative thyroid hormone receptor α1 (TRα1) increased proliferation at postnatal days 0 and 3, adult testicular weight, and sperm reserve. This reproduced the global receptor-knockout phenotype, localizing the effect to Sertoli cells [34]. Triiodothyronine (T3), at concentrations of 10 nM or higher, suppressed FSH-stimulated proliferation in cultured neonatal rat Sertoli cells while increasing differentiation-associated transcripts [35]. These experiments identify a thyroid-dependent limit on Sertoli expansion; its contribution to human NOA remains untested. The final Sertoli-cell population consequently depends on both mitogenic stimulation and the duration of the proliferative period. FSH exposure and a thyroid-dependent stop signal regulate different aspects of that developmental capacity.

### 4.2. LH/hCG–LHCGR Signaling and Sertoli Androgen Competence

LH and hCG activate Leydig-cell LHCGR. In LHβ-null mice, Leydig hypoplasia, reduced steroidogenic-gene expression, and testosterone deficiency accompanied arrest at the round-spermatid stage. hCG restored testosterone and spermatogenic progression [36]. Circulating testosterone nevertheless provides an imperfect surrogate for androgen exposure within the interstitium and seminiferous microenvironment [37].

Human monogenic disease provides complementary loss-of-function evidence. A systematic review included 88 individuals with biallelic inactivating *LHCGR* variants, including 70 with a 46,XY karyotype. Histology predominantly showed absent Leydig cells, with immature cells in some specimens and isolated reports of normal testicular tissue and gonadoblastoma. hCG-stimulated testosterone was low in 24 of 31 tested individuals. At pubertal or postpubertal age, basal testosterone was low in 37 of 39 and LH elevated in 41 of 42. All four men assessed for fertility had oligo- or azoospermia [38].

Among 315 men with otherwise unexplained spermatogenic failure, seven carried heterozygous *NR5A1* missense variants. Six had azoospermia, cryptozoospermia, or severe oligozoospermia, representing approximately 4% of the 149 men in those categories. The remaining carrier had moderate oligozoospermia. All four with endocrine data had low inhibin B; two had testosterone at or below the lower reference limit. Variant proteins reduced transactivation of *CYP11A1* and *AMH* promoters [39]. Because NR5A1 regulates both steroidogenic and Sertoli programs, this genotype does not isolate the lesion to one compartment.

Aromatization modifies local androgen exposure by converting testosterone to estradiol. Human developmental samples from neonates to 60 months showed strong neonatal and infantile Leydig aromatase staining, which declined in older children. Estrogen receptor beta was detectable in Sertoli, interstitial, and germ cells, whereas estrogen receptor alpha was absent or barely detectable [40].

In 76 men with NOA and 18 obstructive azoospermia controls, aromatase localized mainly to Leydig cells, with increased transcript and protein abundance in NOA. Serum and intratesticular testosterone or estradiol concentrations did not correlate. Among 72 untreated NOA participants, strong aromatase staining was more frequent after negative than positive sperm retrieval: 26 of 50 versus 2 of 22 [41]. Four anastrozole-treated men had a higher intratesticular testosterone-to-estradiol ratio than untreated participants, although paired pretreatment samples were unavailable [41]. Increased Leydig CYP19A1 has also been associated with steroidogenic dysfunction in Sertoli-cell-only testes [42].

These associations concern local steroid conversion; they do not establish altered Sertoli AR competence or the direction of causation. Aromatization can reduce AR-ligand availability while supplying estradiol to distinct estrogen-receptor pathways.

The molecular mechanism of androgen-dependent *AMH* repression has been examined in an immortalized prepubertal mouse Sertoli-cell line expressing human AR. Reporter and chromatin-immunoprecipitation experiments indicated that liganded AR represses the *AMH* promoter through steroidogenic factor 1 (SF1) sites, rather than a canonical androgen-response element [43].

Sertoli-selective AR disruption establishes causality in mice, with the affected spermatogenic stage varying between models. One knockout preserved Sertoli number but produced only approximately 3% of normal round-spermatid numbers and no elongated spermatids [44]. A conditional/hypomorphic model instead emphasized defective progression from round to elongating spermatids and impaired spermiation [45]. These results place Sertoli AR activity at several meiotic and postmeiotic steps. Their different arrest phenotypes should therefore be retained when interpreting the dependence of spermatogenesis on Sertoli androgen signaling.

### 4.3. AMH, Inhibin, and Activin

Fetal *AMH* expression begins through the SOX9–SF1–GATA4–WT1 network, largely independently of gonadotropins. Before functional AR acquisition, FSH increases AMH through Sertoli proliferation and expression per cell; subsequently, androgenic repression predominates [46]. Postpubertal AMH therefore remains sensitive to androgen exposure and receptor competence, as well as the underlying Sertoli phenotype. High AMH can accompany deficient androgen exposure or action, persistent immature-like features, or exposure-associated remodeling. Its interpretation gains specificity when paired with AR expression, proliferative status, junctional organization, and germ-cell context.

Inhibin B links Sertoli activity to pituitary FSH feedback. Before spermatogenesis, it reflects Sertoli number and FSH responsiveness; later, germ-cell–Sertoli interactions increasingly influence its concentration. In experimental models, activin can provide a partially opposing local signal through related transforming growth factor β (TGF-β) family receptors. Its effects on proliferation and germ-cell differentiation depend on context. The pubertal atlas found declining Sertoli *INHA*, encoding the inhibin α subunit, and postpubertal Leydig *INHBA* expression, encoding the inhibin βA subunit. Spermatogonia expressed activin receptors. Undifferentiated spermatogonia expressed inhibitors of activin signaling, and tissue staining supported declining *INHA* [23]. These observations identify signaling potential without measuring the assembly, secretion, or activity of mature inhibin or activin dimers.

### 4.4. Growth and Specification Pathways

Sertoli-specific deletion of insulin and IGF1 receptors reduced late fetal and early neonatal proliferation in mice. Adult testis size and daily sperm production fell by approximately 75% [47]. Deletion of the negative regulator *PTEN* restored late fetal proliferation, adult testis size, and sperm output despite the absence of both receptors [48]. This rescue places PI3K–AKT activity downstream of INSR/IGF1R growth signaling and identifies intracellular competence as a requirement for normal FSH-supported expansion. It is also consistent with paracrine insulin-like growth factor (IGF) input from interstitial progenitors, although the rescue did not directly test that source. Endocrine stimulation and local growth signals converge on the same intracellular proliferative machinery.

Canonical WNT4/RSPO1–β-catenin and non-canonical WNT5A signaling have different developmental roles. WNT4/RSPO1–β-catenin activity is associated with the alternative supporting-cell program; human fetal atlases map its spatially and transcriptionally restricted expression [15]. WNT5A instead participates in androgen-dependent Sertoli–spermatogonial communication [49]. In mouse experiments, LH-mediated testosterone suppressed Sertoli *WNT5A* and thereby restrained spermatogonial stem-cell self-renewal [50]. This non-canonical experiment did not establish β-catenin involvement. These distinct findings require WNT effects to be specified by ligand, pathway branch, responding cell, and developmental window. A generic claim of WNT activation would combine mechanisms that can have different effects on somatic specification and germ-cell support.

Fetal Sertoli-derived DHH acts on PTCH1-positive interstitial cells during Leydig specification. *Dhh*-null mice lacked normal adult spermatogenesis. Mechanistic analysis identified impaired acquisition of SF1- and CYP11A1-positive fetal Leydig states, without defective precursor migration, proliferation, or survival [51,52]. DHH therefore specifies an interstitial cell state through Sertoli-to-Leydig communication, rather than acting simply as an additional steroidogenic hormone.

DLK1 expression marks human Leydig states but does not directly measure canonical NOTCH activation [29]. Pubertal ligand–receptor analysis separately identified NOTCH as a candidate pathway in changing niche communication [23]. These observations support investigation of interstitial regulation without establishing a common DLK1–NOTCH mechanism.

### 4.5. Retinoic Acid and Germ-Cell Differentiation

Retinoic acid (RA) links the somatic niche to germ-cell differentiation and meiotic entry. RA–STRA8 signaling triggers meiosis in animal experimental systems. The pubertal atlas detected no *STRA8* transcripts in its 7- and 11-year donor samples, although sparse sampling and transcript dropout limit interpretation [23]. This finding did not implicate RA in the earlier spermatogonial transition observed in those donors. RA activity therefore concerns germ-cell progression within the developing niche, rather than directly defining a mature Sertoli or Leydig state. In this model, niche organization conditions the setting for a germ-cell response, while STRA8 marks a distinct step from earlier spermatogonial differentiation.

## 5. Etiology-Specific Somatic-Cell States in NOA

Similar histological endpoints can arise through different mechanisms. Experiments targeting sex-maintenance genes demonstrate Sertoli-to-granulosa-like transdifferentiation in mice [53]. Comparable lineage evidence is unavailable in human NOA. Etiology-specific findings therefore require interpretation within their developmental and injury context (Table 2).

### 5.1. Idiopathic NOA and Sertoli-Cell-Only Patterns

Zhao et al. examined Sertoli development and disease-associated states in three men with idiopathic NOA, three with Klinefelter syndrome, and one with a Y-chromosome microdeletion. All three idiopathic cases had SCO histology. Their Sertoli cells formed the following two transcriptional groups: one resembled an early developmental state, whereas the other combined a pathological program with features of a later developmental state. WNT-pathway activation and increased proliferative behavior supported the authors’ interpretation of impaired maturation in part of the idiopathic Sertoli population [54]. This study established etiologic heterogeneity and identified a candidate regulatory pathway. Developmental alignment describes molecular resemblance but cannot determine whether the adult phenotype reflects incomplete maturation or later remodeling. Culture perturbations are discussed in Section 7.3.

Alfano et al. profiled three patients with idiopathic germ-cell aplasia and validated selected findings in small independent tissue sets. Leydig cells showed reduced *INSL3* and *HSD17B3* with increased *DLK1*/*NOTCH2*, consistent with an immature-like steroidogenic program. DNA damage, senescence, immune recruitment, and inflammatory signaling accompanied these changes. Collagen I-rich peritubular walls were thickened, whereas basement-membrane collagen IV was depleted [55]. These findings identify coexisting developmental and injury-associated features within the same testes. They support a mixed somatic phenotype without establishing a return from a previously mature Leydig state.

### 5.2. Germ-Cell Maturation Arrest and Mixed Histology

Germ-cell maturation arrest does not establish whether the initiating defect arose in germ cells or their somatic support. Loss of germ-cell signals can remodel the niche, while endocrine or somatic defects can impair germ-cell progression. Etiology-specific mechanisms or longitudinal evidence are needed to distinguish these directions.

A clinical series included five men with NOA and three with obstructive azoospermia (OA). Paired single-cell RNA and chromatin-accessibility profiling in three NOA and two OA testes identified eight Sertoli subclusters and proposed three pseudotime states [56]. The NOA samples differed histologically, including residual spermatocytes, disordered germ cells and sperm, and SCO. Immunofluorescence localized candidate markers, although the three proposed maturity-subtype markers were demonstrated in OA tissue. The study expands the description of Sertoli heterogeneity; its small histological subgroups cannot define a general maturation-arrest trajectory. In cryptozoospermia, a separate study identified altered spermatogonia alongside increased fibrotic peritubular myoid cells, macrophages, vessels, and perivascular T cells [57]. These findings describe a comparator with impaired spermatogenesis. A somatic-state classification specific to maturation-arrest NOA requires joint assessment of arrest stage and somatic features in an adequately replicated cohort.

### 5.3. Klinefelter Syndrome

Klinefelter syndrome combines congenital genotype with pubertal change and progressive tissue injury. Comparative single-cell analysis associated altered Sertoli *XIST* expression and X-linked dosage with interstitial-cell programs [58]. The proposed pubertal sequence was reconstructed across donors; *XIST* expression alone does not establish chromosome constitution.

Winge et al. combined morphology, maturity-marker expression, and chromosome assays to characterize local testicular mosaicism. In adult Klinefelter tissue, mature-marker-positive type-A Sertoli cells were predominantly *XIST*-negative and frequently carried one X chromosome. Immature-like type-B cells usually expressed *XIST* and more often retained two X chromosomes. Focal spermatogenesis was associated with 46,XY Sertoli cells and spermatogonia [59]. A separate series examined 22 individuals classified as non-mosaic by peripheral karyotyping. Postpubertal Sertoli cells surrounding spermatogonia were predominantly 46,XY, whereas comparable prepubertal cells were 47,XXY; postpubertal SCO tubules contained both constitutions [60]. These studies connect local chromosome constitution with Sertoli morphology, maturity markers, and focal germ-cell support. The authors’ chromosome-loss model is biologically plausible, although cross-sectional sampling cannot distinguish a loss event from earlier mosaicism and selective survival or expansion.

Yan et al. investigated the developmental origins of Klinefelter testicular dysfunction. Their fetal single-cell dataset comprised 38,952 cells from two Klinefelter and two control testes at 20–21 weeks postfertilization. A separate adult dataset included two Klinefelter testes and one control. Adult Leydig profiles aligned earlier along a reconstructed developmental trajectory than control profiles. Fetal Klinefelter Leydig cells showed increased progenitor representation, reduced differentiated fetal Leydig representation, and altered steroidogenic markers. Mitogen-activated protein kinase (MAPK) signaling and expression of the X-linked gene *EIF1AX* were increased. Computational analysis suggested reduced Leydig-to-Sertoli midkine signaling, with lower *MDK* and *LRP1* expression confirmed by quantitative polymerase chain reaction. *XIST*-negative Sertoli cells showed abnormal inferred cell-cycle distributions, while immunofluorescence detected reduced ZO-1, β-catenin, and occludin signals [61]. These results establish that somatic abnormalities are already detectable during fetal life. The accompanying perturbations support a role for MAPK in fetal Leydig differentiation (Section 7.3).

The fetal observations address early dysfunction directly and should be interpreted against that objective. They do not require proof of persistent adult immaturity to remain informative. Separate fetal and adult donors cannot establish persistence within a lineage, and junction-associated protein abundance does not measure Sertoli barrier function. Leydig differentiation and Sertoli dysfunction also represent distinct compartment-specific findings; one should not substitute for the other.

Longitudinal endocrine data describe how compartment abnormalities emerge during puberty. A retrospective cohort followed 57 patients with Klinefelter syndrome, aged 9–25 years, from the prepubertal period. Puberty began spontaneously at a mean age of 12.6 years and lasted a mean of 4.1 years. Rising FSH and an inadequate inhibin B response indicated seminiferous-compartment impairment within the first 12–18 months. Inhibin B failed to attain the Tanner stage 5 reference range and fell below 92 pg/mL by two years after onset. AMH declined, although this also occurs during normal androgen-dependent Sertoli maturation. Compensated Leydig insufficiency, defined by elevated LH with low-normal testosterone, emerged after three years. Among 36 participants completing puberty, 34 met the study definition of Sertoli-compartment insufficiency and 29 met its Leydig-compartment definition [62]. Testosterone replacement in 26 of these participants requires treatment-aware interpretation of late-pubertal estimates. This sequence supports earlier seminiferous impairment but does not identify cellular transitions.

Local chromosome constitution and adjacent tubular phenotypes help explain why serum testosterone and whole-biopsy classifications incompletely describe residual competence in Klinefelter testes.

### 5.4. AZF Deletions and Monogenic Spermatogenic Failure

Somatic-state evidence for azoospermia factor (AZF) deletions remains sparse. The single deletion case in Zhao et al. lacked sY84 and sY86, but deletion-extension analysis was unavailable. Retained *UTY* expression cannot define the genomic breakpoint. The finding is therefore described as an AZFa-region deletion of unresolved extent. Sertoli cells showed a relatively adult-like, immune-activated molecular phenotype [54]. This case cannot define the phenotype of complete AZFa deletions or other AZF genotypes.

Sequencing cohorts identify many monogenic causes involving germ-cell processes, including meiotic entry [5]. Such lesions may produce SCO or maturation arrest without primary Sertoli immaturity. Somatic alterations may modify the phenotype or follow germ-cell loss; their initiating role requires evidence of a somatic-cell-autonomous mechanism.

### 5.5. Developmental Endocrine Comparators: Congenital Hypogonadotropic Hypogonadism

In severe untreated CHH, congenital gonadotropin deficiency precedes the adult prepubertal testicular phenotype. This known endocrine deficit makes CHH a developmental comparator for primary testicular NOA. Serial-biopsy and gonadotropin-treatment evidence is evaluated in Section 7.1 [63,64,65].

### 5.6. Cryptorchidism and Acquired Gonadotoxic Injury

Cryptorchidism combines developmental abnormality with thermal and structural injury. Germ-cell loss begins in infancy, can affect the contralateral descended testis, and is more severe with longer undescended duration. Observational studies cannot fully separate duration from underlying disease severity. Orchiopexy corrects location and temperature but may not correct the developmental lesion or replenish lost spermatogonia [66]. Adult bilateral disease is associated with poorer sperm production, lower inhibin B, and higher gonadotropins than unilateral disease. These systemic findings do not distinguish residual Sertoli immaturity from dysfunction after germ-cell loss or injury.

Chemoradiotherapy provides an acquired comparator when a previously functioning testis encounters a defined insult. Germ cells are generally more sensitive than Leydig cells. Alkylating regimens can cause prolonged azoospermia, whereas Leydig injury usually requires higher radiation exposure. Recovery depends on the agent, cumulative dose, fractionation, surviving spermatogonia, and follow-up duration [67]. These observations demonstrate compartment-selective loss. They do not establish somatic-cell reversion, although inflammation, barrier disruption, fibrosis, or endocrine failure may follow germ-cell depletion. An SCO endpoint after gonadotoxic treatment consequently differs mechanistically from developmental germ-cell absence.

### 5.7. Immune, ECM, and Vascular Remodeling

The inflammatory and fibrotic findings in idiopathic germ-cell aplasia illustrate how injury can accompany immature-like Leydig features (Section 5.1) [55]. Human tissue and cell experiments provide a possible link between immune signaling and ECM composition. Decorin deposition increased in germ-cell arrest and SCO with fibrosis, and tumor necrosis factor α increased decorin production by peritubular cells from fibrotic testes [68]. These results support an interaction between inflammation and structural remodeling without identifying decorin as the initiating lesion.

Vascular and perivascular changes also accompany impaired spermatogenesis [57]. In Klinefelter tissue, computational analysis linked endothelial–Leydig communication with ECM-related programs [58]. Peripheral-blood and semen profiling in a broader infertility cohort included 12 men with NOA, with blood T-cell sequencing in six. NOA was associated with T-cell senescence signatures and inflammatory mediators [69]. Because testicular somatic cells were not profiled, these findings establish a systemic association rather than a local causal mechanism.

Aging provides another comparator for adult somatic dysfunction. A study of three younger and three older donors, with validation in ten donors per group, identified somatic stress, impaired Leydig redox homeostasis, and reduced pleiotrophin signaling [70]. A lifespan atlas of 214,369 cells from 35 men aged 21–69 years identified age-associated peritubular remodeling, altered Leydig steroid metabolism, and macrophage immune programs [71]. Peritubular differences were prominent in donors in their fourth decade, while Leydig and macrophage changes characterized donors in their sixth decade. These cross-sectional differences support age matching of adult controls. They do not demonstrate sequential changes within individuals. Varicocele is associated with lower testosterone, which may rise after repair, but its Sertoli states remain inadequately mapped [72]. Neither an age-associated transcriptional shift nor a postoperative endocrine response establishes a distinct NOA maturity phenotype.

Etiology changes the interpretation of similar biopsy patterns. Developmental endocrine deficiency, local chromosome mosaicism, and acquired gonadotoxic loss provide different explanations for residual somatic function. Comparisons therefore require compartment-specific findings alongside the relevant developmental and injury context (Table 2).

**Table 2:** Somatic findings across NOA etiologies and comparator settings.

Condition or Comparator	Principal Somatic Finding	Interpretation
Idiopathic NOA and germ-cell aplasia [54,55]	Heterogeneous immature-like programs occur with variable inflammation and structural injury.	Molecular phenotype and injury should be described separately; SCO does not define one state.
Klinefelter syndrome [58,59,60,61,62]	Fetal studies show abnormalities; separate postnatal samples reveal spatial differences in somatic markers, chromosome constitution, and tissue integrity.	Developmental disturbance and local heterogeneity are supported; cellular history requires additional temporal evidence.
AZF deletions and monogenic disease [5,54,73,74]	Somatic evidence varies by genotype; many diagnosed defects primarily affect germ-cell processes.	Genotype and germ-cell pattern cannot substitute for direct somatic characterization.
Severe CHH: endocrine comparator [31,63,64,65,75]	The under-stimulated compartment responds to gonadotropins; serial biopsies show forward Sertoli maturation.	Responsiveness to replacement of a missing signal does not establish reversal of primary testicular NOA.
Cryptorchidism and gonadotoxic injury [66,67]	Developmental or acquired lesions combine with variable germ-cell loss and tissue damage.	Residual somatic maturity, injury, and functional reserve must be assessed individually.
Androgen deprivation and gender-affirming hormone therapy (GAHT): exposure comparators [76,77,78]	Adult-exposure groups show altered Sertoli programs and accompanying niche changes.	Exposure associations do not demonstrate the previous cellular baseline or recovery after withdrawal.
Aging and varicocele: adult comparators [70,71,72]	Stress, inflammatory, vascular, or endocrine changes are reported.	An endocrine change alone cannot establish restoration of local niche function.
End-stage tubular sclerosis [6,14]	Architectural loss dominates the sampled tissue.	The endpoint does not identify the starting somatic state or the sequence of injury.

The full condition- and model-level synthesis is provided in Supplementary Table S4. Comparator findings do not establish the same mechanism or treatment response in primary testicular NOA. States can vary within one biopsy.

## 6. Interpreting Maturity, Injury, and Cellular History

The biological question determines which evidence is needed. A molecular signature can identify a state-associated program, whereas a claim about tissue competence requires structural or functional support. Developmental history and acquired exposure introduce a separate temporal question. The proposed framework records these dimensions within a named Sertoli or Leydig compartment, retaining the local tissue context (Figure 4).

### 6.1. Phenotype, Injury, and Chronology

A molecular signature and a functional measurement answer different questions about the same compartment. Their agreement can strengthen characterization, while disagreement can identify a biologically informative dissociation. Neither pattern establishes when the state arose. The reporting framework retains those distinctions rather than compressing them into a single maturity score.

Hormonal exposure, genotype, and local germ-cell survival can alter the interpretation of similar marker patterns. Neighboring tubules may retain different degrees of mature organization and function. A diagnosis therefore need not occupy one fixed position in the framework.

Injury should be described using the features actually assessed, including DNA damage, inflammation, extracellular-matrix changes, vascular abnormalities, senescence, or sclerosis. Each finding is recorded as detected, not detected in the assessed material, or not assessed. An immature-associated signal does not indicate low injury, and extensive fibrosis does not establish prior maturity. Their coexistence in germ-cell aplasia and Klinefelter tissue illustrates why these observations should remain separately visible [55,59]. Separate recording does not assert that injury and maturity are biologically independent. The two are often correlated, and shared mechanisms are plausible; the separation keeps the measured feature distinct from the interpretation.

Etiology helps explain how a lesion may begin, without specifying every subsequent somatic response. Complete AZFa and AZFb deletions carry different germ-cell and retrieval implications from partial deletions and other genotypes [73]. Clinical exome findings further separate germ-cell defects from broader developmental and endocrine mechanisms [5,74]. Somatic changes should therefore be interpreted alongside genotype and tissue distribution.

### 6.2. A Proposed Criterion for Stronger Maturity Characterization

The following four domains can contribute to a maturity interpretation: molecular state, compartmental morphology or architecture, receptor or secretory competence, and relevant function. For Sertoli cells, functional evidence may concern barrier integrity or germ-cell support; for Leydig cells, it may concern steroidogenesis. Directional agreement across two distinct domains, including a non-transcriptomic domain, is proposed as a practical benchmark for stronger characterization. This benchmark is a reporting proposal, not an established threshold or a prerequisite for a useful molecular study. Two domains are the smallest number that can show non-redundant agreement, and a non-transcriptomic domain is required because transcript abundance cannot demonstrate structure, competence, or function. The compartment constraint prevents evidence from one cell type supporting a claim about another. These choices are reasoned rather than empirically derived, and other thresholds remain defensible. Descriptors are applied within an etiology-defined group rather than across NOA as a whole, following the organization of Table 2 and Supplementary Table S4.

The principle of combining markers with morphology and function also appears in stem-cell characterization standards [79]. Its transfer to the testicular niche requires explicit biological definitions. RNA and same-marker protein abundance measure related molecular features and should not automatically count as independent domains. By contrast, quantitative junctional continuity, polarity, ligand-responsive output, or steroid secretion add a different type of observation. Evidence should concern the same compartment and distinguish within-donor concordance from convergence across different donor subsets. Some observations touch more than one domain; in situ marker localization, for example, reports molecular state and spatial organization together. Each observation should be assigned to a single domain, and that assignment should be stated.

Some boundaries remain uncertain. Retained prepubertal-type cords, together with immature markers, provide architectural evidence relevant to Sertoli organization [13]. However, cord or lumen formation also reflects germ-cell and other niche contributions. This observation should be described directly, with its tissue-level interpretation stated, rather than excluded to produce a negative study verdict. Prospective applications should define architectural readouts before assessment and test agreement between observers. Reproducible classification would still require independent validation against biological outcomes.

The required evidence also changes with the claim. A chronological transition requires temporal information, while true dedifferentiation additionally requires lineage continuity and coordinated reversal toward an earlier state. Rodent somatic-cell fate experiments illustrate possible plasticity but do not establish this sequence in adult human NOA [53,80]. The selected studies are therefore presented through supported findings and remaining questions, without a pooled pass-or-fail conclusion.

### 6.3. Contributions of Single-Cell and Spatial Methods

Single-cell RNA sequencing resolves cellular programs that bulk tissue averages obscure. Single-nucleus methods can improve recovery of fragile or large cells, while chromatin assays identify candidate regulatory regions. These methods describe distinct molecular properties and do not directly measure receptor activity or cellular function. Age-dependent adult references are also needed because the somatic niche varies across the reproductive lifespan [27,71]. Supplementary Table S3 records study designs and donor groups.

Spatial methods connect molecular profiles with anatomical neighborhoods. Testicular Slide-seq mapped cellular classes and seminiferous-tubule organization at approximately 10 μm resolution [81]. Integrated transcriptomic, chromatin, spatial, and histological observations in androgen-deprived testes further associated hormonal suppression with altered Sertoli and interstitial features [77]. Such integration can localize a phenotype and suggest mechanisms for testing. Its contribution should be assessed against those aims before considering questions that require a different design.

Pseudotime orders molecularly related cells along a model-dependent path; it does not measure elapsed time or trace a lineage [82]. RNA velocity estimates transcriptional direction under additional assumptions. Ligand–receptor co-expression nominates possible interactions, but secretion, receptor engagement, and biological effects require direct testing. These computational approaches generate mechanistic hypotheses that can guide targeted experiments.

### 6.4. Limitations of the Evidence and This Synthesis

Small donor cohorts limit generalization even when many thousands of cells are profiled. Donors remain the biological replicates, and donor-aware pseudobulk or suitable mixed models are needed to address differential-expression uncertainty [83]. This concern is especially relevant when new disease samples are compared with reused public controls. Where the proposed criteria are unmet, this reflects what the available studies measured rather than the biology of the compartment. Most designs were not built to characterize maturity across several domains.

Control provenance can also influence the apparent state. Healthy reference tissue, obstructive azoospermia, and tissue adjacent to other pathology are not interchangeable [9]. Age, hormonal exposure, and tissue collection should be considered alongside etiology. Dissociation, filtering, and cell recovery can alter the observed composition or induce stress programs. Integration may leave technical variation or remove biologically meaningful differences. Spatial measurements may combine signals from more than one cell type.

Cross-sectional tissue comparisons cannot distinguish a persistent state from an acquired change without additional historical evidence. Germ-cell depletion and altered endocrine exposure may affect both molecular and functional measurements. Culture experiments provide direct evidence of responsiveness, but culture itself can modify cell state. These design limits qualify the stronger inference; they do not negate the original molecular or experimental finding.

This synthesis adds its own limitations. PubMed and reference tracing do not guarantee comprehensive coverage, and the worked examples were chosen purposively. The extraction was verified against the primary sources by a second author, but it was not reclassified independently, and the proposed criterion lacks external validation. The selected examples therefore support an interpretive discussion rather than estimates of literature frequency, diagnostic performance, or clinical utility. Table 3 lists these examples with additional evidence that stronger claims would require.

## 7. Evidence for Reversibility and Niche-Directed Intervention

A treatment response can indicate pathway activity, developmental progression, or recovery of a lost function. These outcomes require different evidence. Forward maturation follows replacement of a missing developmental signal; reversal requires restoration toward a prior adult state after correction of an acquired lesion. A claim of reversibility should specify the intervention and the molecular, architectural, endocrine, or reproductive endpoint being restored.

### 7.1. Hormone-Dependent Development in CHH

Dwyer et al. enrolled 18 men with severe CHH and prepubertal testes in a randomized open-label study. Thirteen were analyzed after five exclusions for nonadherence or GnRH nonresponse. Four months of rFSH priming increased inhibin B, testicular volume, and Sertoli- and spermatogonial-cell numbers. Serial biopsies showed cytoskeletal rearrangement, Sertoli polarization, and tight-junction development before pulsatile GnRH treatment. During subsequent GnRH therapy, sperm appeared in all seven primed men and four of six controls. Maximal sperm count and time to first sperm did not differ significantly [63]. The serial histological and endocrine changes support hormone-dependent forward maturation. The small analyzed sample limits comparisons of fertility outcomes between regimens.

Other gonadotropin studies establish clinical responsiveness across different developmental settings. In a retrospective two-center cohort of 35 adolescents and young adults, 19 completed treatment and 18 provided semen samples; sperm was detected in 15 [64]. The cohort included congenital and acquired, complete and partial gonadotropin deficiency, without an untreated or testosterone-only comparator. A review of 11 nonrandomized infant studies included 71 boys. Among 85 cryptorchid testes assessed in six studies, 62 showed partial or complete descent; pubertal fertility outcomes were unavailable [65]. In another retrospective cohort, gonadotropin treatment produced ejaculated sperm in 27 of 35 azoospermic men with hypogonadotropic hypogonadism. Microdissection retrieval succeeded in seven of eight persistently azoospermic men, and 18 achieved biological fatherhood [75]. Larger baseline testicular volume was associated with ejaculated-sperm response in an unadjusted comparison involving only eight nonresponders. This observation cannot validate preserved architecture as a response predictor. Biological fatherhood also depends on partner factors and assisted reproduction. These clinical outcomes complement the serial-biopsy evidence but do not establish reversal of genetic or fibrotic primary testicular NOA.

### 7.2. Androgen Deprivation and GAHT as Exposure Comparators

Multimodal profiling compared four androgen-deprived participants with six controls. Treated testes showed fewer adult-associated Sertoli features, more immature-like programs, lower Leydig representation, and immune and ECM differences [77]. GnRH-based prostate-cancer treatment suppressed LH and FSH alongside androgen exposure. The cross-sectional design therefore cannot isolate androgen effects or determine recovery after withdrawal.

Among 106 orchiectomy specimens after GAHT, 99 contained tubules co-expressing SOX9, AR, and AMH; 90 showed this pattern exclusively. Mixed patterns often included fibrosis or hyalinization. Transcriptomic profiling involved six treated samples and developmental controls [76]. The study identifies an exposure-associated immature-like phenotype. Combined treatment exposures and absent baseline tissue leave acquisition and reversibility unresolved. Recovery after withdrawal of exogenous testosterone or anabolic-androgenic steroids documents endocrine-dependent spermatogenesis in selected men but does not define somatic-cell state transitions [84].

### 7.3. Human Ex Vivo Pathway Perturbation

In the Zhao study, ICG-001 changed maturity-associated markers and AMH secretion in cultured Sertoli cells and improved feeder support of mouse germline stem cells [54]. This demonstrates functional modulation in culture. Isolation had already induced proliferative, immature-like features in adult control cells, so restoration of the human seminiferous niche cannot be inferred. In the Yan study, MAPK inhibition reduced proliferation and increased testosterone secretion in isolated fetal Klinefelter Leydig cells. After seven days in fetal-tissue culture, the ratio of NR2F2-positive progenitors to CYP11A1-positive fetal Leydig cells decreased [61]. *EIF1AX* overexpression in a mouse Leydig cell line increased proliferation and reduced steroidogenic gene expression and CYP11A1 protein. These perturbations support a role for MAPK and *EIF1AX* in fetal Leydig dysfunction. A similar isolated-cell response occurred in controls, and the experiments did not test reversal of adult Klinefelter NOA.

### 7.4. Organoids and Organotypic Culture

Three-dimensional systems preserve more tissue organization than isolated-cell cultures. Cryptorchid infant tissue maintained tubules for 60 days with increased Sertoli AR and reduced AMH, while spermatogonia declined and barrier organization remained abnormal [85]. Organoids from one 46,XY and two Klinefelter donors produced testosterone and expressed postmeiotic transcripts. Cells were classified as haploid using fluorescence in situ hybridization limited to chromosomes X, Y, and 18 [86]. These readouts require confirmation of complete meiosis, recombination, imprinting, genome-wide chromosome constitution, and reproductive competence before clinical translation.

### 7.5. The Boundary with Clinical Hormonal Optimization

Primary-NOA intervention trials should distinguish etiology and baseline maturity-associated features from tissue injury. Where ethical and feasible, paired sampling should relate changes in somatic state to endocrine function and reproductive outcomes.

A single-center retrospective analysis evaluated 1256 selected men with NOA undergoing micro-TESE, including 601 with Klinefelter syndrome and 655 with a 46,XY karyotype. Fertility-preserving hormonal therapy increased serum testosterone but did not improve sperm-retrieval rates in either group; overall retrieval was 32.4% [87]. Nonrandom treatment allocation and heterogeneous regimens permit residual confounding. Nevertheless, biochemical improvement did not indicate improved spermatogenesis. Two weak 2026 European Association of Urology recommendations advise against hormonal stimulation before TESE outside trials and against routine pretreatment to improve sperm recovery in hypergonadotropic NOA [4]. Treatment of symptomatic hypogonadism is a separate indication. A reproductive benefit from hormonal optimization in primary testicular NOA therefore remains unproven.

## 8. Translational Implications and Research Priorities

### 8.1. Clinical Interpretation

A somatic-cell profile may eventually supplement histology by linking etiology to local tissue competence. Clinical use first requires evidence that the profile adds information beyond established clinical and genetic predictors. Spatial heterogeneity also matters: a whole-biopsy average may conceal a small region with residual germ-cell support.

Seminal proteins and extracellular-vesicle RNA offer accessible candidates, but the cited discovery studies have not established externally validated clinical prediction [88]. Evidence for salvage micro-TESE predictors also derives mainly from retrospective cohorts [89]. A tissue signature would need an accessible correlate, a prespecified threshold, and validation against retrieval outcomes before clinical adoption. The proposed descriptors are intended to improve how studies report maturity evidence. Any clinical application is a separate step and requires prospective validation against retrieval or treatment outcomes.

### 8.2. Studies Needed to Test the Framework

The first requirement is an etiology-resolved human reference with independent donors and standardized clinical, hormonal, and treatment histories. Molecular profiling should be paired with spatially annotated histology and compartment-specific architectural or functional readouts. Relevant reference groups should remain identifiable rather than being pooled as normal controls. Independent observers should assess the same prespecified features before associations with outcomes are tested. Prospective testing of the descriptors requires a held-out set of studies, application by at least two independent raters, and agreement reported as a raw percentage and kappa before discussion. Comparison with an expert consensus classification would show whether the descriptors capture accepted judgments. The number of studies depends on the expected agreement and the precision sought, so it should be fixed in advance rather than assumed.

Temporal questions require paired observations around a defined perturbation, where repeated sampling is ethical and feasible. Patient-derived organotypic systems can then test specific pathways while retaining more of the local niche. Suitable endpoints include barrier function, steroid secretion, and germ-cell support. Claims of gametogenesis additionally require meiotic, genomic, and functional assessment [90].

A published protocol proposes gonadotropin treatment in hypogonadal idiopathic NOA classified as APHRODITE Groups 3–4 [91]. This endocrine-based selection does not test the somatic reporting framework. Future interventional studies could assess whether baseline tissue features modify response, using prespecified reproductive outcomes and matched molecular or functional measures. Biomarker studies should progress from analytical validation to blinded external validation and assessment of added clinical value.

## 9. Conclusions

Hormonal regulation establishes and maintains somatic-cell functions through developmental windows, receptor competence, and interactions within the testicular niche. In NOA, immature-like molecular programs, structural injury, and retained mature features can occur in different combinations. Their interpretation should preserve the distinction between the phenotype measured and the history inferred. Recording maturity-associated evidence, injury, and chronology separately offers a framework for this purpose. Its proposed criteria require reproducibility testing and biological validation. Existing developmental and experimental studies identify hormone-responsive processes, but their translation to primary testicular NOA depends on etiology and tissue context. Clinical use requires prospective evidence that these observations improve prediction or treatment decisions.

## Figures and Tables

**Figure 1:**
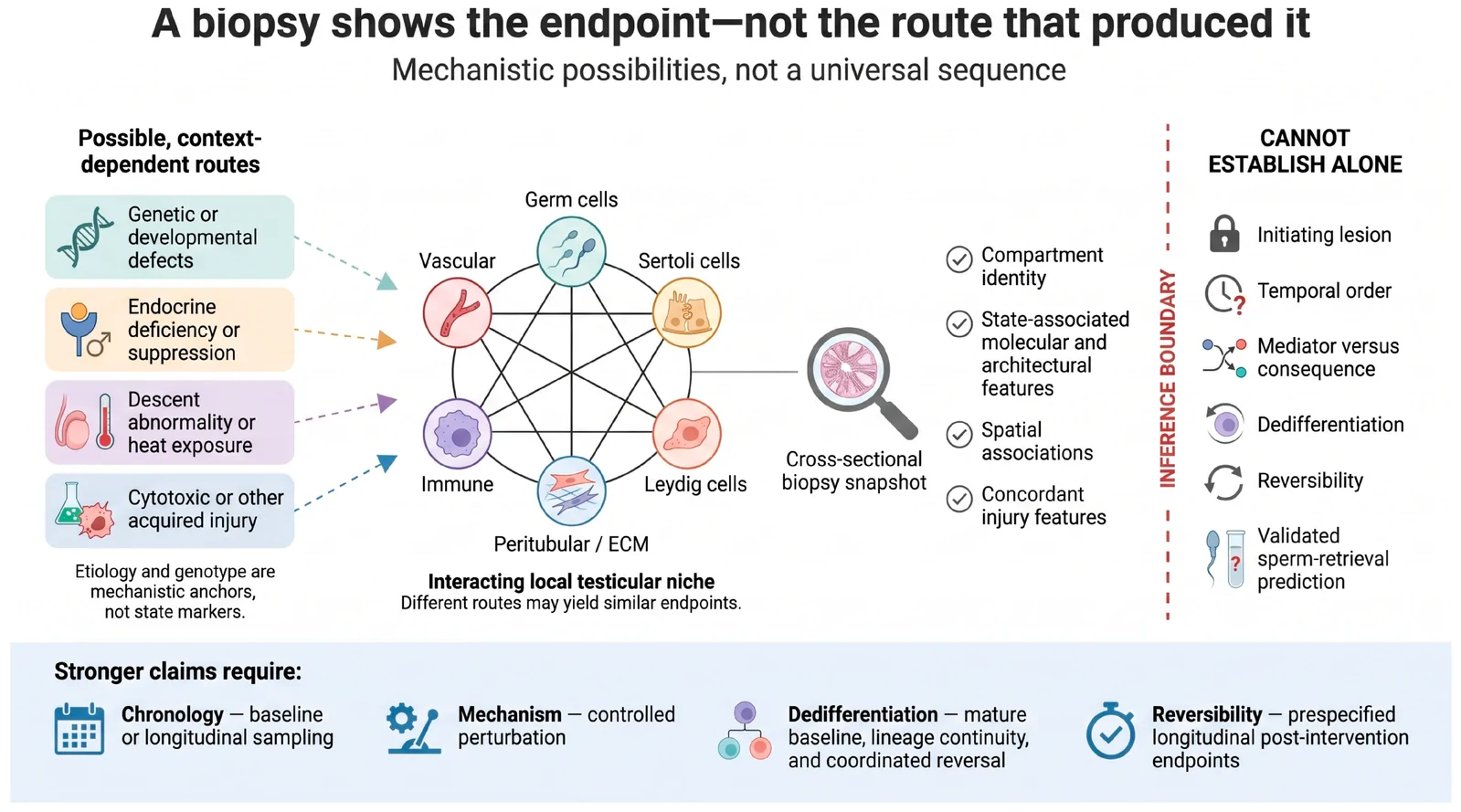
Interpretation of a cross-sectional testicular biopsy. Different initiating conditions can produce similar tissue endpoints through interactions among germ cells and the local somatic niche. A biopsy can describe molecular features, tissue architecture, spatial relationships, and injury in the sampled material. The diagram separates these observations from stronger claims concerning cause, temporal order, lineage change, reversibility, and clinical prediction. Dashed arrows indicate possible context-dependent routes. The additional designs needed for stronger claims are shown below the inference boundary. Abbreviations are defined in the text and the abbreviation list. Created in BioRender. Kaltsas, A. (2026) https://BioRender.com/07fu0uo.

**Figure 2:**
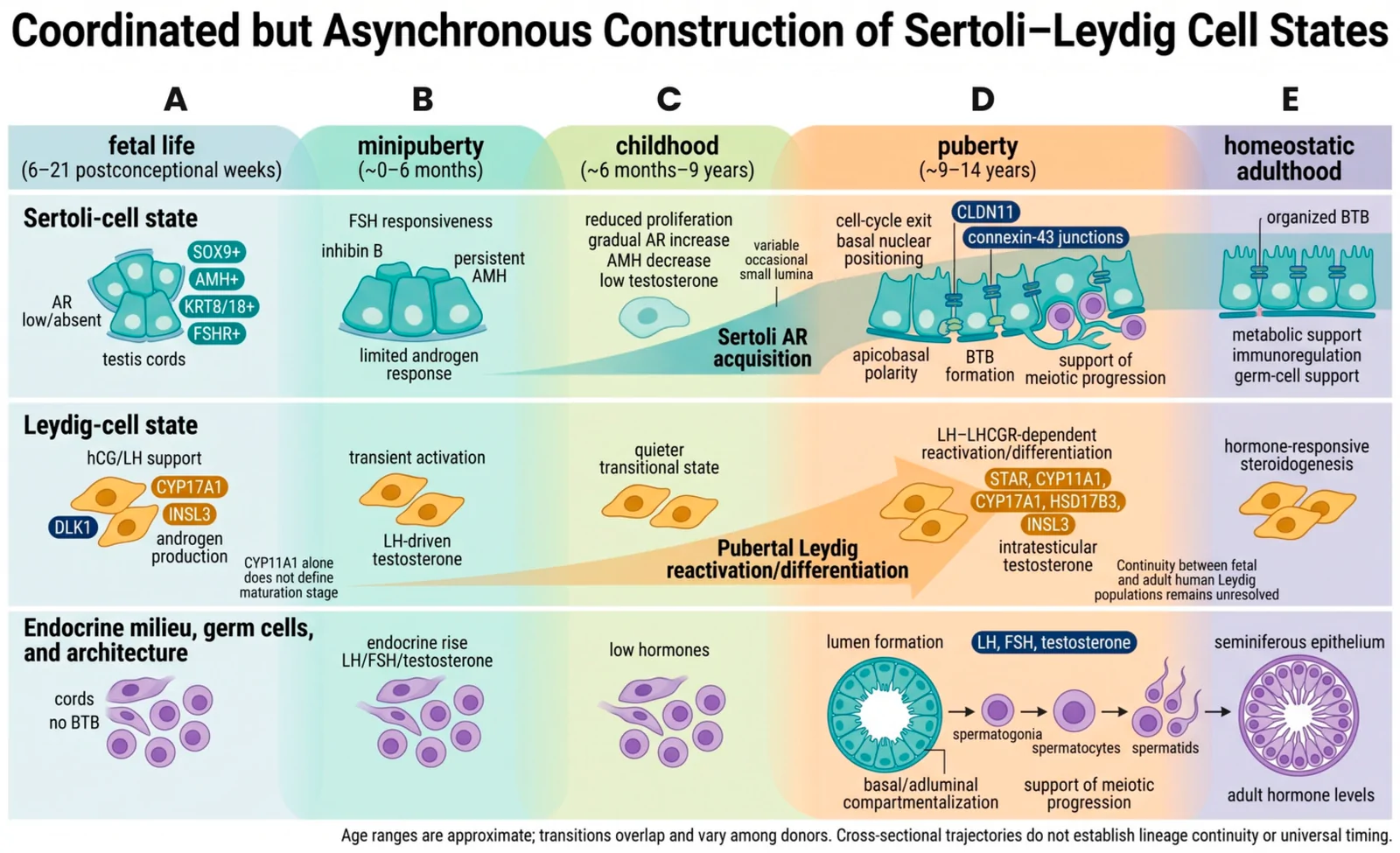
Hormonal development of the testicular somatic compartments. Panels (**A**–**E**) correspond to fetal life, minipuberty, childhood, puberty, and homeostatic adulthood. The rows show Sertoli cells, Leydig cells, and the endocrine and germ-cell context. Sertoli androgen-receptor competence develops gradually, allowing androgen exposure to support pubertal changes in proliferation, polarity, and barrier organization. Leydig steroidogenesis also changes across developmental windows, but several steroidogenic markers occur in fetal and adult populations. Marker patterns therefore require interpretation with age, morphology, and functional evidence. Age ranges are approximate; human fetal-to-adult Leydig lineage continuity remains unresolved. Abbreviations are defined in the text and the abbreviation list. Created in BioRender. Kaltsas, A. (2026) https://BioRender.com/uqnzwzx.

**Figure 3:**
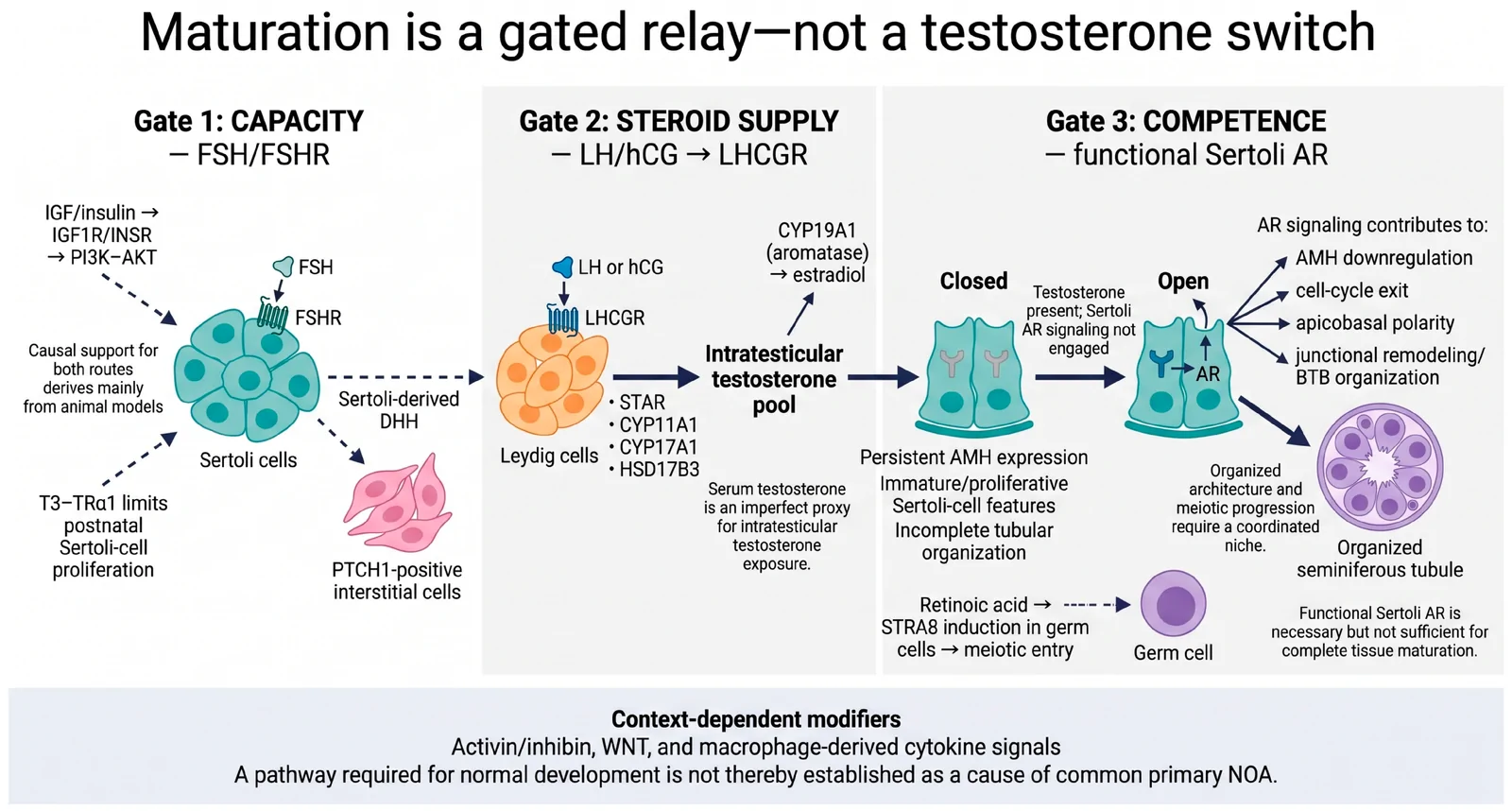
Hormonal and paracrine requirements for somatic-cell maturation. FSH–FSHR signaling supports Sertoli-cell capacity, while LH/hCG–LHCGR signaling maintains Leydig steroid production. Testosterone contributes to Sertoli maturation after androgen-receptor competence develops. The diagram shows dependencies with overlapping developmental timing. IGF/insulin and thyroid signaling modify proliferation; DHH acts on interstitial cells, while retinoic acid supports germ-cell meiotic entry. Solid arrows identify the principal hormonal relationships. Dashed arrows indicate contextual interactions or pathways supported mainly by animal experiments. These relationships do not establish a causal sequence in human NOA. Abbreviations are defined in the text and the abbreviation list. Created in BioRender. Kaltsas, A. (2026) https://BioRender.com/633mnf6.

**Figure 4:**
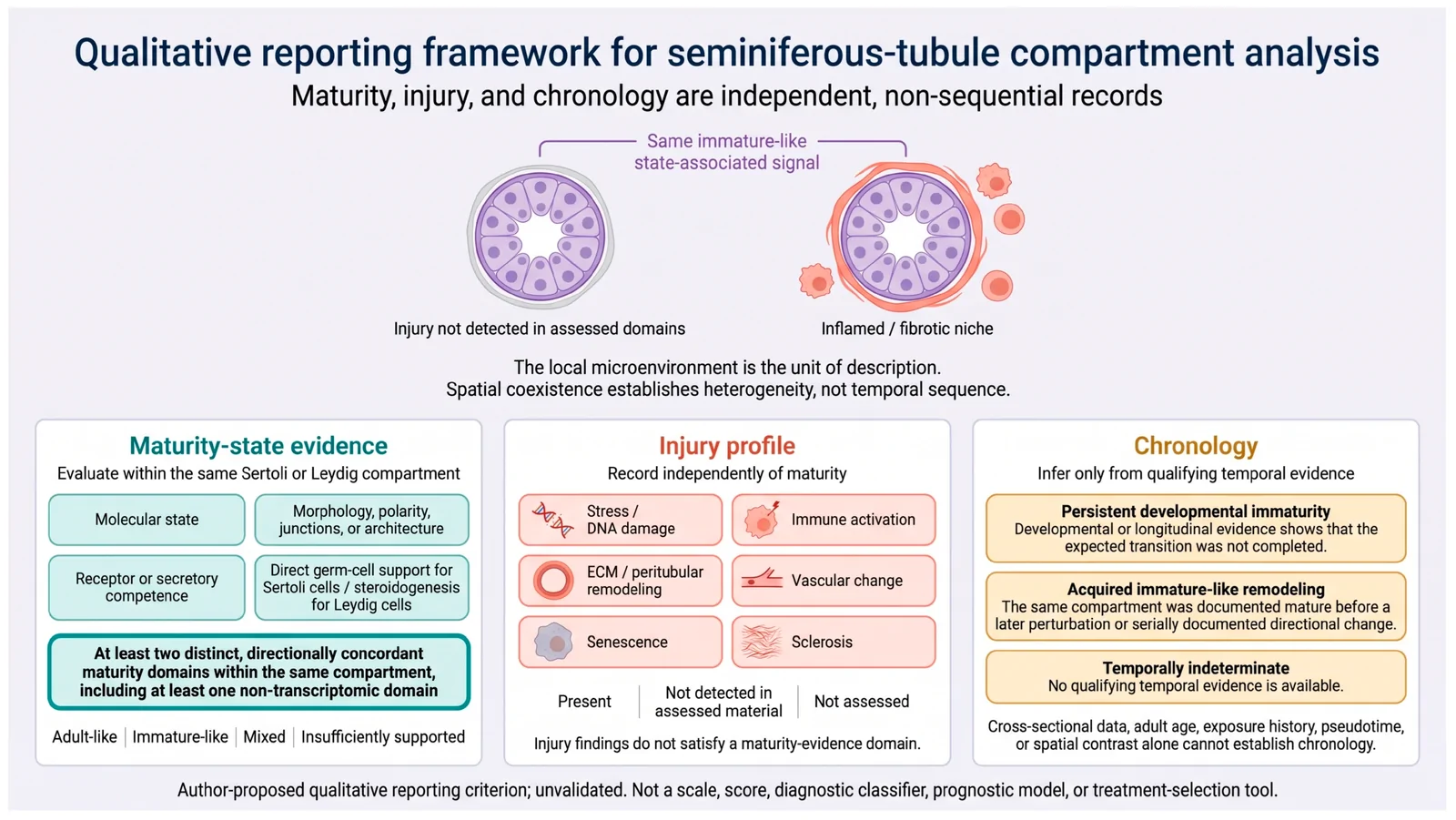
Proposed framework for recording maturity-associated evidence, injury, and chronology. The hypothetical tubules illustrate how similar molecular signals can occur in different injury contexts. Separate records do not imply biological or statistical independence. The two-domain criterion is an unvalidated proposal for strengthening an adult-like or immature-like maturity characterization. Mixed evidence and insufficient support describe unresolved interpretations; they are not study-quality verdicts. Molecular findings remain informative when another domain was not measured. Injury observations cannot substitute for maturity evidence, and chronology requires appropriate temporal data. Abbreviations are defined in the text and the abbreviation list. Created in BioRender. Kaltsas, A. (2026) https://BioRender.com/l3yx4fy.

**Table 1:** Consolidated terminology for somatic-cell states and their interpretation.

Term	Meaning	Interpretive Limit
NOA	Azoospermia attributed to impaired sperm production.	Semen findings alone do not distinguish obstructive from non-obstructive disease.
SCO and germ-cell maturation arrest	SCO denotes sampled tubules without germ cells. Maturation arrest denotes a defined limit of germ-cell progression.	Neither histological pattern defines somatic-cell maturity.
Primary and secondary SCO	Proposed failure of initial germ-cell establishment, or germ-cell loss after establishment, respectively.	Cross-sectional histology does not establish the temporal sequence.
Immature-like molecular program	Measured expression resembles an earlier developmental reference.	This supports a molecular description, without requiring an unmeasured architectural or functional domain.
Persistent developmental immaturity	The expected developmental transition was not completed.	Requires developmental or longitudinal evidence beyond molecular resemblance.
Acquired immature-like remodeling	Mature features decline and development-associated features appear after a documented mature state and subsequent change.	Adult age or exposure history alone does not document prior cellular maturity.
Mature dysfunction	Mature characteristics are retained while a relevant function is impaired.	A mature-marker profile alone does not establish functional competence or dysfunction.
True dedifferentiation	A differentiated cell reverses toward an earlier state.	Requires lineage continuity and coordinated temporal evidence; pseudotime alone is insufficient.
Adult-like or immature-like characterization	Directionally concordant maturity evidence in a named compartment.	Two distinct domains, including a non-transcriptomic domain, are proposed for stronger characterization; the criterion is unvalidated.
Mixed evidence	Spatially resolved or domain-specific features support different descriptions.	Report the distribution and disagreement rather than averaging the whole biopsy.
Insufficiently supported interpretation	The evidence does not resolve the specific broader maturity interpretation.	This describes a claim, not study quality. Findings outside that claim retain their value.
Temporally indeterminate	The history of the observed state is unresolved.	Use when qualifying temporal evidence is unavailable.

Biological and temporal terms describe the tissue or its history. The evidence descriptors summarize the support for a particular interpretation and do not assess overall study quality.

**Table 3:** Selected study contributions and the additional evidence needed for stronger claims.

Study and Compartment	Study Question	Supported Knowledge	Limit of Inference and Additional Evidence
Steger et al., 1999 [13]—Sertoli	Characterize pathological Sertoli marker patterns.	Distinct marker patterns were observed. Immature markers accompanied retained prepubertal cords in one pattern.	This supports an immature tissue phenotype. Temporal evidence is required to distinguish developmental persistence from acquired change.
Zhao et al., 2020 [54]—Sertoli	Map development and NOA states; test WNT inhibition.	Etiology-associated molecular states and improved mouse-germ-cell feeder support after culture perturbation were reported.	Culture altered the starting state. Uncultured architecture and human functional endpoints would be needed to assess coordinated in vivo rescue.
Alfano et al., 2021 [55]—Leydig	Characterize idiopathic germ-cell aplasia.	An immature-like Leydig molecular program coexisted with DNA damage, inflammation, fibrosis, and senescence.	The study separates state-associated signals from injury. Matched Leydig function and temporal evidence would clarify their relationship.
Yan et al., 2026 [61]—Leydig and Sertoli, considered separately	Investigate fetal Klinefelter abnormalities and candidate mechanisms.	Fetal Leydig differentiation-associated changes responded to MAPK inhibition in experimental tissue. Sertoli molecular and junction-associated protein abnormalities were observed.	These findings concern early disease biology. Longitudinal, compartment-specific observations would be needed to test persistence into adulthood.
Dwyer et al., 2013 [63]—Sertoli	Test rFSH priming before GnRH in severe CHH.	Treatment-linked endocrine and serial structural changes support forward Sertoli maturation.	CHH is a developmental endocrine comparator. The findings do not demonstrate reversal of primary testicular NOA.
Guo et al., 2026 [77]—Sertoli	Characterize testicular changes during androgen deprivation.	Molecular and tissue findings support an exposure-associated phenotype, with separate functional experiments identifying candidate responsive processes.	There was no pretreatment or withdrawal sampling. Functional assays in the exposure population would strengthen attribution and recovery claims.

The complete study-by-compartment extraction is provided in Supplementary Table S2. Each example is interpreted in relation to its original purpose; additional requirements apply to the stronger claim shown.

## Data Availability

No new data were created or analyzed in this study. Data sharing is not applicable to this article.

## Supplementary Materials

The following supporting information can be downloaded at https://www.mdpi.com/article/10.3390/cimb48100970/s1.

## Abbreviations

The following abbreviations are used in this manuscript:

AKT	protein kinase B
AMH	anti-Müllerian hormone
APHRODITE	Addressing male Patients with Hypogonadism and/or infeRtility Owing to altereD, Idiopathic TEsticular function
AR	androgen receptor
AZF	azoospermia factor
AZFa	azoospermia factor a region
AZFb	azoospermia factor b region
BTB	blood–testis barrier
CHH	congenital hypogonadotropic hypogonadism
CLDN11	claudin 11
CYP11A1	cytochrome P450 11A1
CYP17A1	cytochrome P450 17A1
CYP19A1	cytochrome P450 19A1 (aromatase)
DHH	desert hedgehog
DLK1	delta-like non-canonical Notch ligand 1
DNA	deoxyribonucleic acid
ECM	extracellular matrix
EIF1AX	eukaryotic translation initiation factor 1A X-linked
FSH	follicle-stimulating hormone
FSHR	follicle-stimulating hormone receptor
GAHT	gender-affirming hormone therapy
GATA4	GATA-binding protein 4
GnRH	gonadotropin-releasing hormone
hCG	human chorionic gonadotropin
HSD17B3	17β-hydroxysteroid dehydrogenase type 3
IGF	insulin-like growth factor
IGF1	insulin-like growth factor 1
IGF1R	insulin-like growth factor 1 receptor
INHA	inhibin subunit alpha
INHBA	inhibin subunit beta A
INSL3	insulin-like factor 3
INSR	insulin receptor
KRT8/18	keratin 8/18
LH	luteinizing hormone
LHCGR	luteinizing hormone/choriogonadotropin receptor
LRP1	low-density lipoprotein receptor-related protein 1
MAPK	mitogen-activated protein kinase
MDK	midkine
micro-TESE	microdissection testicular sperm extraction
NOA	non-obstructive azoospermia
NOTCH	notch receptor
NOTCH2	notch receptor 2
NR5A1	nuclear receptor subfamily 5 group A member 1 (steroidogenic factor 1)
OA	obstructive azoospermia
PCW	postconceptional weeks
PI3K	phosphoinositide 3-kinase
PTCH1	patched-1
PTEN	phosphatase and tensin homolog
RA	retinoic acid
rFSH	recombinant follicle-stimulating hormone
RNA	ribonucleic acid
RSPO1	R-spondin 1
SANRA	Scale for the Assessment of Narrative Review Articles
SCO	Sertoli-cell-only
SF1	steroidogenic factor 1
SOX9	SRY-box transcription factor 9
SRY	sex-determining region Y
STAR	steroidogenic acute regulatory protein
STRA8	stimulated by retinoic acid 8
T3	triiodothyronine
TESE	testicular sperm extraction
TGF-β	transforming growth factor β
TRα1	thyroid hormone receptor α1
UTY	ubiquitously transcribed tetratricopeptide repeat containing, Y-linked
WNT	Wingless-related integration site
WNT4	Wnt family member 4
WNT5A	Wnt family member 5A
WT1	Wilms tumor 1
XIST	X-inactive specific transcript
ZO-1	zonula occludens 1
